# Inhibitory neurotransmission in frontostriatal circuitry: implications for psychomotor and vegetative symptoms in depressive episodes

**DOI:** 10.1186/s12888-026-08573-1

**Published:** 2026-08-31

**Authors:** Linda Steinholtz, Jonas Persson, Elin Thörnblom, Anders Wall, Mark Lubberink, David Fällmar, Robert Bodén

**Affiliations:** 1 Department of Medical Sciences, Uppsala University, Uppsala, Sweden; 2 Department of Surgical Sciences, Molecular Imaging and Medical Physics, Uppsala University, Uppsala, Sweden; 3 Department of Surgical Sciences, Neuroradiology, Uppsala University, Uppsala, Sweden

**Keywords:** Bipolar disorder, Major depressive disorder, Basal ganglia, Terminal insomnia

## Abstract

**Background:**

Gamma-aminobutyric acid (GABA) dysfunction has been implicated in depression, although research findings remain inconsistent. This may indicate that distinct biological mechanisms underlie different symptom profiles in depressive episodes. Altered GABA function might be especially relevant in cases characterised by psychomotor retardation and vegetative symptoms, which are prominent in melancholia. This study aimed to investigate if such melancholic features are related to GABA levels or GABA_A_-receptor availability.

**Methods:**

This was a secondary analysis of data obtained from a randomised controlled trial. Forty-two patients with either bipolar or unipolar depression were assessed before and after a course of repetitive transcranial magnetic stimulation. GABA and glutamate levels in the dorsal anterior cingulate cortex (dACC) were measured using a [^1^H] magnetic resonance spectroscopy MEGA-PRESS sequence. A subset of 28 patients underwent [^11^C]flumazenil positron emission tomography (PET) to evaluate GABA_A_-receptor availability in the dACC, basal ganglia, and hypothalamus. Psychomotor retardation was assessed with the expression subscale of the Clinical Assessment Interview for Negative Symptoms. Sleep disturbances were evaluated using components of the Pittsburgh Sleep Quality Index, while appetite loss was measured using an item from the Montgomery-Åsberg Depression Rating Scale – self-rated. The scores were standardised and combined to create composite measures for vegetative and somatic symptoms. Additionally, baseline psychomotor activity was measured using accelerometry.

**Results:**

The findings did not establish that psychomotor or vegetative symptoms were associated with GABA levels or GABA_A_-receptor availability in the frontostriatal pathways or hypothalamus.

**Conclusions:**

To better understand the relationship between GABA dysfunction and melancholic features, future studies should ideally focus on patients exhibiting more pronounced psychomotor and vegetative symptoms and examine additional cortical regions.

## Introduction

Current knowledge indicates that several functional brain circuits are involved in depressive episodes, with the heterogeneous symptoms of depression possibly reflecting distinct neurobiological mechanisms [1]. One proposed mechanism underlying depression is altered gamma-aminobutyric acid (GABA) neurotransmission, the brain's primary inhibitory signalling system [2].

Post-mortem studies have demonstrated a decreased density of GABA interneurons in the dorsolateral prefrontal and occipital cortices of depressed patients [3, 4]. Further, magnetic resonance spectroscopy (MRS) meta-analyses have reported lower levels of GABA in anterior medial frontal cortex regions in unipolar depression [5]. Only a few studies have compared differences in GABA_A_-receptor availability between depressed patients and healthy controls. A single-photon emission computed tomography (SPECT) study using [^123^I]iomazenil found no differences [6], while a [^11^C]flumazenil positron emission tomography (PET) study reported reduced GABA_A_-receptor binding in the bilateral temporal cortices in depressed patients [7]. However, a recent multimodal study using both GABA MRS and [^11^C]flumazenil PET in the same cohort as this study found no group differences [8]. Notably, none of these studies examined depression subtypes or specific symptoms of depression.

These heterogeneous findings may reflect differing underlying pathologies across different symptom profiles in depression. GABA deficiency may be particularly relevant in psychomotor retardation and vegetative symptoms, both prominent features of melancholia [9, 10]. Previous studies report lower cerebrospinal fluid GABA levels in melancholic depression compared with both healthy controls [11] and other depression types [12]. Further, an MRS study of the occipital lobe showed larger and more consistent GABA reductions in melancholic than in atypical or undifferentiated depression [13].

Psychomotor activity is regulated and coordinated in the basal ganglia, a central functional unit mainly composed of inhibitory circuits [14]. The proposed ‘anterior cingulate loop’, a cortico-subcortical circuit within the basal ganglia, receives projections from the anterior cingulate cortex (ACC), where MRS studies have shown reduced GABA levels in depressive disorders [5]. Vegetative symptoms of depression often include diurnal mood variation, terminal insomnia, weight loss, and reduced appetite [9, 10]. The suprachiasmatic nucleus in the hypothalamus serves as the master circadian pacemaker in mammals, while the basal ganglia contribute to the sleep-wake cycle [15, 16].

Several of the above-mentioned regions have been connected to psychomotor symptoms in depression [17]. In late-life depression, melancholic psychomotor dysfunction has been linked to reduced striatal volume [18, 19], while improvements in melancholic-related psychomotor symptoms following ECT have been associated with increased basal ganglia volume [20]. Reduced frontostriatal regional cerebral blood flow (rCBF) [21, 22] and aberrations in thalamocortical connectivity [23] have also been reported in depression with psychomotor retardation.

Considering the above, GABA dysfunction in depression may be particularly associated with psychomotor retardation and vegetative symptoms, potentially involving frontostriatal circuits such as the anterior cingulate loop, of which the dorsal ACC (dACC) is a key node involved in cognitive processing of emotions [1]. However, this relationship remains largely unexplored.

The aim of this study was to investigate whether psychomotor retardation and vegetative symptoms during depressive episodes are related to GABA levels in the dACC and GABA_A_-receptor availability in the dACC, basal ganglia, and hypothalamus. In addition to cross-sectional associations, we examined whether longitudinal changes in symptom severity corresponded to changes in GABA measures. We hypothesised that greater psychomotor and vegetative symptom severity would be associated with lower GABA levels and reduced GABA_A_-receptor availability.

## Methods

### Participants and procedures

This study was a secondary analysis of data from 42 participants in a randomised clinical trial of repetitive transcranial magnetic stimulation at the psychiatric clinic of Uppsala University Hospital [24]. The aim of the clinical trial was to evaluate intermittent theta burst transcranial magnetic stimulation (iTBS) for anhedonia, avolition, and blunted effects in schizophrenia and depression. To be included, patients had to be between 18 and 59 years old, experiencing a current bipolar or unipolar depressive episode confirmed by the Mini International Neuropsychiatric Interview (MINI) (Swedish version 6.0.0), have had stable psychiatric medication for the past month, and score ≤ 40 on the Motivation and Pleasure Scale-Self-Report [25, 26]. Exclusion criteria included a diagnosis of epilepsy, the presence of ferromagnetic or other metal implants, benzodiazepine treatment, substance use disorder (excluding nicotine and caffeine), and pregnancy. No participant had a depressive episode with mixed features or a history of rapid cycling. All pharmacotherapy was maintained throughout the study period. A transition to a hypomanic or a manic state during the clinical trial was considered an adverse event leading to discontinuation, although no such event occurred.

Examinations conducted during the clinical trial were used to investigate whether changes in GABA were associated with changes in psychomotor and vegetative symptoms [24]. All examinations were performed on the weekday before treatment initiation and included clinical evaluations, accelerometry, magnetic resonance imaging (MRI), and MRS. Additionally, a subsample of 28 patients underwent positron emission tomography (PET). All assessments, except for accelerometry, were repeated four weeks later, following 10–15 consecutive weekdays of twice-daily active or sham iTBS. Notably, this study focused on the covariation over time between GABA measurements and symptoms, disregarding treatment effects.

### Clinical assessments

To assess psychomotor retardation, the expression subscale of the Clinical Assessment Interview for Negative Symptoms (CAINS) was utilised. CAINS measures the severity of negative symptoms and includes two main domains across two subscales: (i) motivation and pleasure in social, vocational, and recreational life and (ii) emotional expression and speech [27]. The expression subscale contains four items: facial expression, vocal expression, expressive gestures, and quantity of speech. Rating is based on observation of behaviour during the interview and ranges from 0 to 16, with higher scores denoting a more severe psychomotor impairment. Originally developed to assess negative symptoms in schizophrenia and schizoaffective syndrome, the CAINS has also been validated for use in depressive episodes [28]. In the present study, the expression subscale was used as an observational measure for psychomotor impairment, and will hereafter be referred to as the *psychomotor score*.

All patients completed the Pittsburgh Sleep Quality Index (PSQI), a questionnaire designed to assess sleep quality over the past month [29]. To evaluate the patients’ sleep patterns with a focus on terminal insomnia, we selected two subparts from the PSQI: component 4, which measures the percentage of time spent asleep relative to time in bed, and item 5b, which assesses the frequency of waking up during the night or in the early morning. The scores from these selected PSQI components were summed, resulting in a total score ranging from 0 to 6, with higher scores indicating more severe sleep disturbances.

Additionally, patients completed the self-rated version of the Montgomery-Åsberg Depression Rating Scale (MADRS-S), which evaluates the severity of depression symptoms [30]. Item 4, reflecting self-rated appetite reduction, was used as a pragmatic indicator of appetite loss within the constraints of the available dataset, with scores ranging from 0 to 6. As this single item is not a dedicated appetite instrument, it was interpreted only as one component of a broader vegetative symptom construct.

Baseline scale scores were standardised using z-transformation and then summed to create composite scores, with each sub-score given equal weight. Composite score changes were calculated by z-transforming the difference between follow-up and baseline values for each component and then summing the resulting standardised values.

In this way, two composite scores were created based on the scores above. The first combined appetite reduction and sleep disturbance into a *vegetative score*. The second composite score also included psychomotor ratings from the CAINS expression subscale, providing a more comprehensive assessment of somatic dysfunction, referred to as the *somatic score*.

### Accelerometry

An accelerometer measures physical activity, providing an objective estimate of motor symptoms in depressive episodes [31]. This study utilised the two-electrode Firstbeat Bodyguard 2 (Firstbeat Technologies Ltd., Jyväskylä, Finland), which was attached to the chest. The device was applied in the morning and worn by patients for at least 24 h. It sampled data at a frequency of 12.5 Hz for each dimension, with an 8-bit resolution. Peak values in all three directions—anteroposterior, mediolateral, and vertical—were attained at a force of 4G. The acceleration data were transformed into a vector magnitude, and an algorithm developed from ActiGraph accelerometers was used to compute activity counts using Matlab R2017b scripts [32].

The mean activity count per minute between 12 noon and 6 pm was selected to measure the physical activity level. This time frame was identified within the first ~ 24 h of monitoring, even if the patient wore the accelerometer for a longer time. Night-time and morning data were excluded to avoid inaccurate activity readings due to potential sleep disturbances. Additionally, previous research has shown that motor activity varies most in the afternoon between different depression subtypes [33].

### Magnetic resonance imaging and spectroscopy

All subjects were scanned with a 3 Tesla Achieva dStream scanner (Philips Healthcare, Best, The Netherlands) equipped with a 32-channel head coil. Structural T1-weighted images of the brain were obtained using a 3D TFE sequence featuring a TR/TE of 8.2/3.8 ms, an 8° flip angle, a 256 × 256 mm field of view, and a spatial resolution of 1 × 1 × 1 mm. A board-certified neuroradiologist (DF) visually assessed all morphological images to rule out anatomical malformations and incidental findings.

During the same scanning session, MRS was conducted using MEGA-PRESS: a J-difference Mescher–Garwood spectral editing technique integrated within a point-resolved spectroscopy sequence [34]. The experimental parameters were set to TR/TE 2000/68 ms, with a spectral bandwidth of 2000 Hz, 1024 data points, and a phase cycling of 4. Data acquisition was organised into 40 groups, each starting with an unsuppressed water line, followed by four consecutive pairs of water-suppressed transients in ON and OFF conditions. This resulted in a total of 160 averages for each condition. The unsuppressed water lines were used to correct magnetic field drift and update the carrier frequency of the radiofrequency pulses in each group.

The spectroscopic volume of interest consisted of a single voxel measuring 40 × 40 × 20 mm (left-right x anterior-posterior x feet-head directions). This voxel was positioned centrally over the midline, comprising the bilateral cingulate gyrus. Its lower boundary aligned with the upper border of the corpus callosum, and its front boundary was in line with the most anterior part of the corpus callosal genu.

The MEGA-PRESS spectra were pre-processed, modelled, and quantified using Osprey 2.5.0 in Matlab R2023b [35]. This process involved alignment using the probabilistic spectral registration algorithm and correction for eddy currents. After phase and frequency corrections, the individual averaged spectra were visually inspected for irregularities. The spectra were then fitted using the Osprey linear combination method with the default basis set, performing separate fittings for the difference and OFF spectra while employing a baseline knot spacing of 0.55 ppm [36]. The unified segmentation algorithm in Statistical Parametric Mapping 12 (SPM12; Wellcome Trust Centre for Neuroimaging, University College London, UK) was utilised for tissue segmentation of the T1-weighted images into grey matter, white matter, and cerebrospinal fluid.

**Fig. 1:**
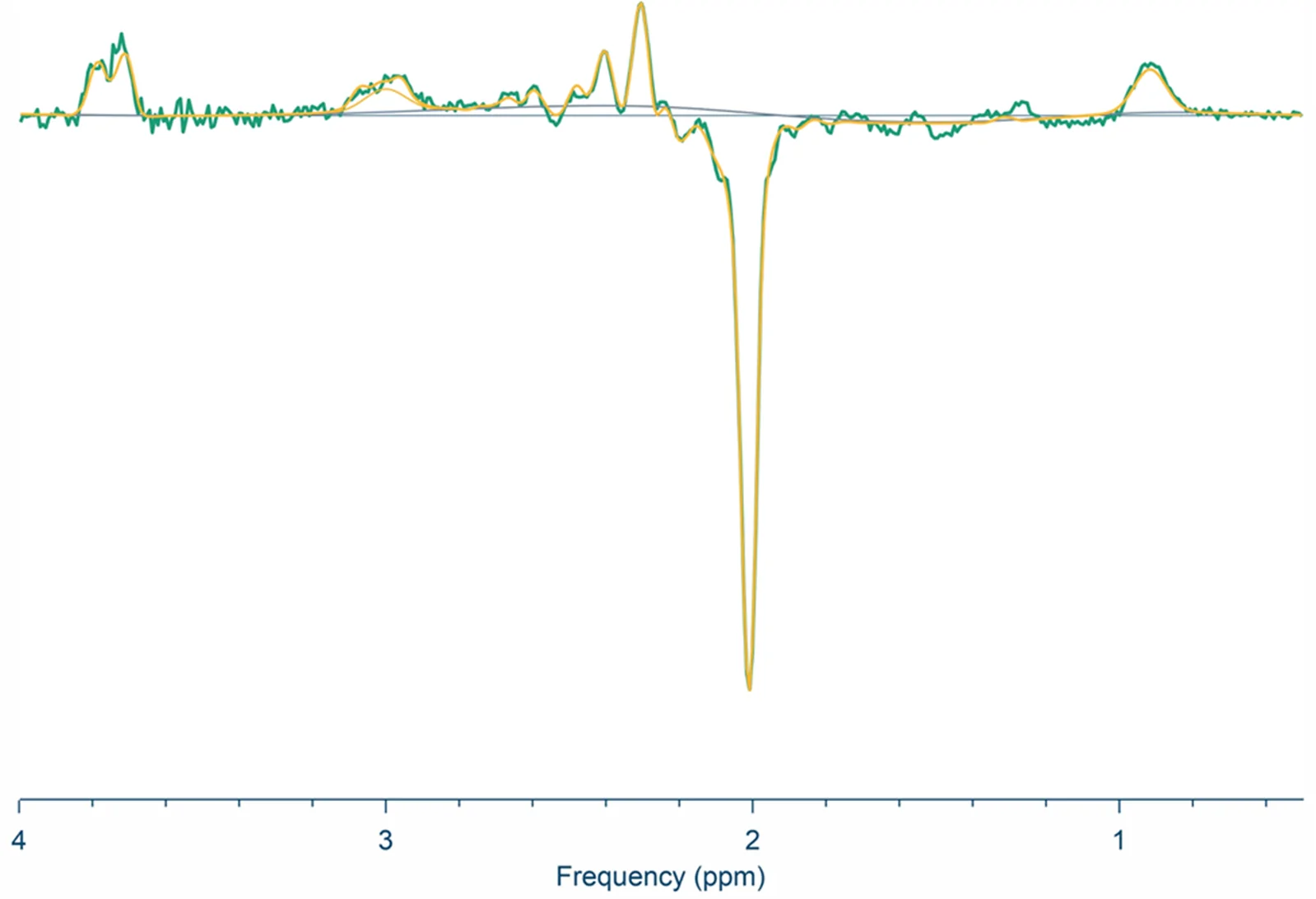
Difference (‘ON-OFF’) spectra (green) and model fit (yellow) example

The difference (ON-OFF) spectra were used to quantify GABA (Fig. 1), and the levels were estimated with alpha-corrected tissue water as a reference [37, 38]. The average signal-to-noise ratio (SNR) and the mean full width at half maximum (FWHM) of creatine were calculated as quality metrics for the MRS signal. To assess the quality of the fit, relative residuals were calculated by comparing the amplitude of the residuals to the standard deviation (SD) of the noise.

### Positron emission tomography

PET measurements were conducted using a Discovery MI PET/computed tomography (CT) system (GE Healthcare, Waukesha, WI). A low-dose CT scan was obtained for attenuation correction (120 kV, 10–20 mA, noise index 170). The patients received an intravenous injection of [^11^C]flumazenil (2–4 MBq/kg body weight) at the beginning of the dynamic PET scan (4 × 15 s, 4 × 60 s, 2 × 150 s, 2 × 300 s, 2 × 600 s, total duration 40 min) [39]. PET images were reconstructed using time-of-flight ordered subsets expectation maximisation with three iterations and 34 subsets, incorporating resolution recovery and a 3-mm Gaussian post-processing filter. The synthesis of [^11^C]flumazenil followed previously described methods [39].

All dynamic PET images were corrected for within-scan movement using VOIager 4.0.7 (GE Healthcare) software. T1-weighted MR images were segmented into grey and white matter, following co-registration with a summation of the initial five minutes of the PET scan, using Statistical Parametric Mapping 8 (SPM8; Wellcome Trust Centre for Neuroimaging, University College London, UK). With the centrum semiovale white matter serving as the reference region, parametric images of the non-displaceable binding potential (BP_ND_), an estimate of GABA_A_-receptor availability, were created via a basis function implementation of the simplified reference tissue method [7, 40, 41]. This reference region was defined by globally eroding the two outer layers of voxels from the cranial half of each subject’s segmented white matter image, resulting in an average volume of 4.4 ± 1.4 cm³ across the sample. To obtain a reference tissue time-activity curve, the reference region was projected over all frames of the dynamic PET scan.

Additional image preprocessing was performed using SPM12 within MATLAB 2019b. This process involved aligning the BP_ND_ images with the T1-weighted image through co-registration. The T1-weighted images underwent tissue classification, bias correction, and spatial normalisation using SPM12’s unified segmentation algorithm. The resulting deformation fields were then applied to transform both the individual T1-weighted and BP_ND_ images into the Montreal Neurological Institute (MNI) space. Finally, the BP_ND_ images were smoothed using a kernel with a FWHM of 8 × 8 × 8 mm.

**Fig. 2:**
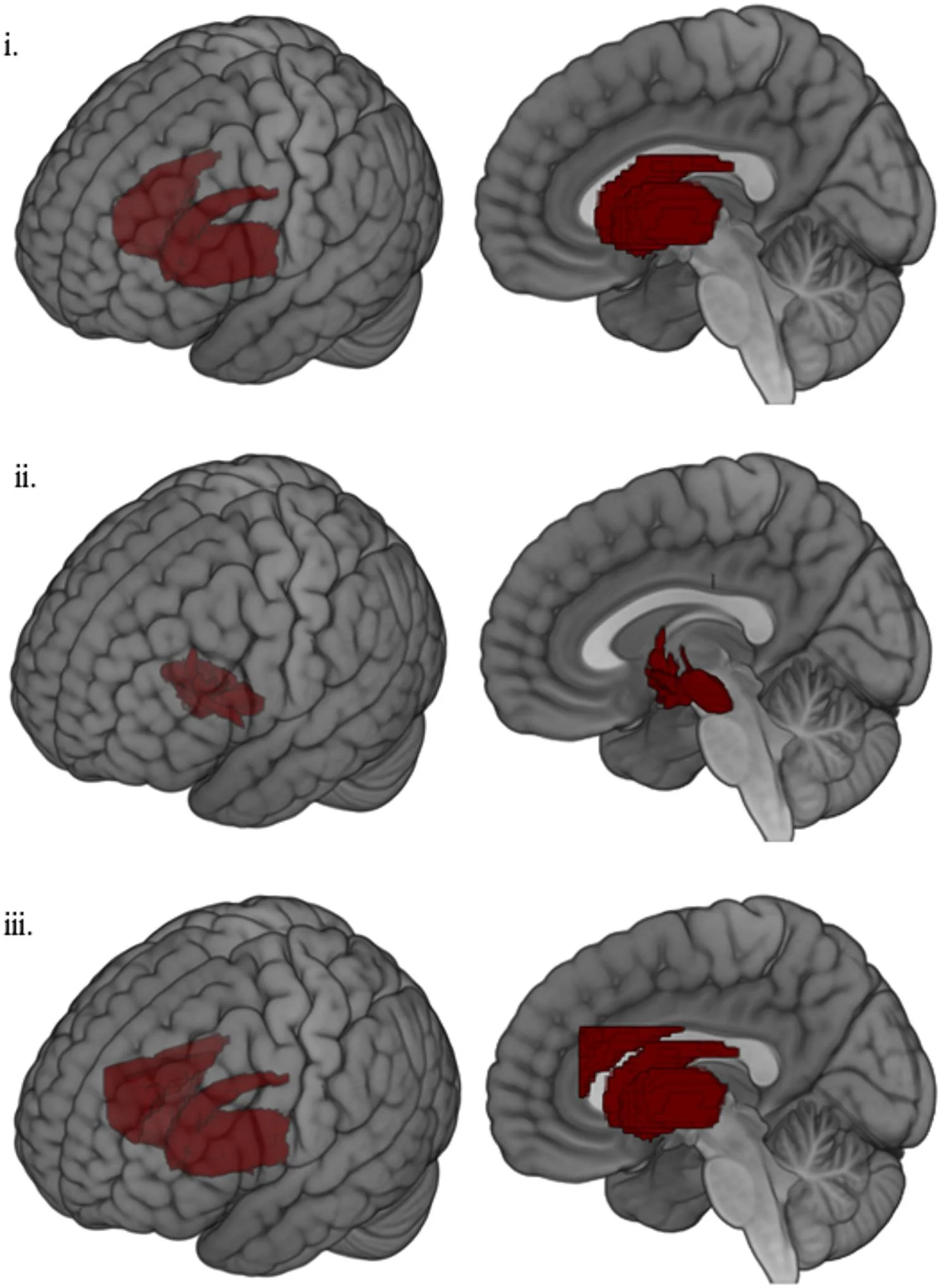
Regions of interest. From the top: (**i**) the striatum (nucleus accumbens and caudate nucleus) and the pallidum, (**ii**) the hypothalamus, and (**iii**) the dorsal anterior cingulate cortex, striatum, pallidum, and the hypothalamus

Three regions of interest (ROI) were used (Fig. 2). The first region included basal ganglia areas involved in psychomotor activity: the striatum (nucleus accumbens and caudate nucleus) and the pallidum, defined using the AAL3 atlas [42]. The second region was the hypothalamus, defined by the NSD atlas [43]. Lastly, the third region comprised the dACC, nucleus accumbens, caudate nucleus, pallidum, and the hypothalamus, constituting the anterior cingulate loop. The definitions for this region were derived from a combination of the AAL3 atlas and the NSD atlas [14, 42, 43]. 

The inverse deformation fields from the previous pre-processing steps were applied to align the atlas region with each participant’s subject space, creating individual masks. These masks were then used to extract the individual mean BP_ND_ within the volumes of interest.

### Statistical methods

All baseline assessments (*psychomotor score*, *vegetative score*, *somatic score*, and accelerometry metrics) were correlated with GABA levels in the dACC. The *psychomotor score* at baseline and the accelerometry measurement were correlated with mean BP_ND_ in the basal ganglia ROI. The *vegetative score* at baseline was correlated with mean BP_ND_ in the hypothalamus ROI. Correlations were computed using Kendall’s Tau b. This non-parametric measure was chosen due to the limited sample size and the presence of tied ranks, as it provides a robust rank-based measure of association that is less sensitive to distributional assumptions.

Longitudinal associations were examined by correlating changes in scores from baseline to follow-up (not available for accelerometry) with corresponding changes in GABA levels and BP_ND_. Linear mixed-effects models were initially explored but yielded singular fits, likely reflecting the limited variability in the data and the availability of only two observations per participant. Therefore, change scores were analysed using Kendall’s Tau-b.

The change metrics were also incorporated into multiple regression models, with changes in GABA levels and BP_ND_ as the dependent variables and changes in clinical scores as predictors. Additionally, treatment allocation was included as a categorical predictor to account for the effects of iTBS.

The baseline *somatic score* and accelerometry metrics were also analysed in relation to BP_ND_ using voxel-wise regression within the anterior cingulate loop–hypothalamus ROI. Parametric PET images were entered into a voxel-wise general linear model in SPM, using the ROI as an inclusive mask, with the *somatic score* and accelerometry metrics serving as regressors in separate analyses. F-contrasts were defined to assess voxels where BP_ND_ was related to the *somatic score* and accelerometry, respectively. Voxels were considered significant at a cluster threshold of pFWE < 0.05, adjusted for mask volume, using a cluster-forming threshold of *p* < 0.001, uncorrected.

Similarly, changes in *somatic scores* between baseline and follow-up were analysed using voxel-wise regression. This analysis was conducted on PET images representing differences in BP_ND_ between baseline and follow-up parametric PET images. Additionally, treatment allocation was included as a covariate.

The analyses were performed in JASP 0.19.1 [44] and SPM12 in MATLAB 2023b.

## Results

One participant did not have baseline MRS measurements. Baseline neurotransmitter levels could not be estimated for five participants due to missing water references, which were attributed to software issues during acquisition in four cases and poor data quality in one case. MRS data at follow-up were missing for two participants, and two additional participants lacked a water reference. In the PET subsample, post-treatment PET data were unavailable for four participants.

**Table 1:** Demographic and clinical data at baseline and change from baseline

	Entire sample		PET subsample	
	*n* = 42		*n* = 28	
**Age (years)**,** median (q1**,** q3)**	27 (22, 34)		26 (22, 34)	
**Range (years)**	18–54		18–54	
**Men/women**	21/21		15/13	
**Bipolar depression**,** n**	3		2	
**Pharmacotherapy**				
Antidepressants, n	36		23	
Antipsychotics, n	8		5	
Antiepileptics, n	5		2	
	**Baseline**	**Change**	**Baseline**	**Change**
**MADRS-S baseline**,** median (q1**,** q3)**	31 (24, 35)	-4 (-8, 0)^a^	32 (24, 35)	-4 (-8, 0)
**CAINS expression**,** median (q1**,** q3)**	7 (5, 10)	-1 (-2, 1)^a^	8 (5, 10)	-1 (-3.5, 0)
**Sleep summation score**,** median (q1**,** q3)**	4 (2, 5)^b^	0 (-1, 0)^c^	3 (2, 5)	0 (-1, 0)
**Appetite score**,** median (q1**,** q3)**	2 (1, 2)	0 (-1, 0)^a^	2 (1, 3)	0 (-1, 0)
**Accelerometry (CPM)**,** median (q1**,** q3)**	354 (290, 562)^a^	-	361 (278, 571)	-

CAINS: Clinical Assessment Interview for Negative Symptoms, CPM: counts per minute, MADRS-S: Montgomery Åsberg Depression Rating Scale – self-rated, PET: positron emission tomography, q: quartile

^a^ n = 41

^b^ n = 40

^c^ n = 36

Table 1 presents demographic and clinical data for the entire sample, as well as the subsample that underwent PET. Table 2 details GABA levels and GABA_A_-receptor binding potentials. Overall, mean changes between baseline and follow-up measurements were minimal for both clinical assessment scores and GABA measures.

**Table 2:** Gamma-aminobutyric acid (GABA) levels and [^11^C]flumazenil BP_ND_: baseline and change from baseline

	Baseline	Change
	*n* = 36	*n* = 33
**GABA level (i.u.)**,** mean (SD)**	2.2 (0.6)	0.1 (0.9)
	*n* = 28	*n* = 24
**[** ^**11**^ **C]flumazenil BP**_**ND**_ **, mean (SD)** *Basal ganglia*	39.3 (5.7)	0.0 (4.1)
**[**^**11**^**C]flumazenil BP**_**ND**_, **mean (SD)** *Hypothalamus*	5.3 (0.8)	0.0 (0.6)

BP_ND_: non-displaceable binding potential, GABA: gamma-aminobutyric acid, i.u.: institutional units, SD: standard deviation

Correlation analyses of baseline *psychomotor scores* revealed no association with GABA levels in the dACC or mean BP_ND_ in the basal ganglia ROI (Table 3). Similarly, changes in *psychomotor scores* did not correlate with changes in GABA measures (Table 4). Accelerometry also showed no correlation with GABA levels in the dACC or mean BP_ND_ in the basal ganglia ROI (Table 3).

**Table 3:** Correlations between melancholic features and gamma-aminobutyric acid (GABA) measures at baseline

	GABA level (i.u.)		[^11^C]flumazenil BP_ND_	
	Kendall’s τb	*p*	Kendall’s τb	*p*
			*Basal ganglia*	
**Psychomotor z-score**	0.07	0.6	0.02	0.9
**Accelerometry**,** CPM**	0.03	0.8	-0.21	0.1
			*Hypothalamus*	
**Vegetative z-score**	-0.14	0.1	-0.21	0.1
**Somatic z-score**	-0.10	0.4		

BP_ND_: non-displaceable binding potential, CPM: counts per minute, GABA: gamma-aminobutyric acid, i.u.: institutional units

**Table 4:** Correlations and multiple regression models of change from baseline in *psychomotor*, *vegetative*, and *somatic scores* and gamma-aminobutyric acid (GABA) measures

	GABA level change (i.u.)						[^11^C]flumazenil BP_ND_ change					
	correlation		regression model*			correlation			regression model*			
	Kendall’s τb	*p*	F	df	*p*	Kendall’s τb		*p*	F	df	*p*	
**Change from baseline**:						*basal ganglia*						
** - Psychomotor z-score**	0.03	0.8	0.78	2, 30	0.5	-0.21		0.2	1.74	2, 21	0.2	
						*hypothalamus*						
*** - *** **Vegetative z-score**	-0.13	0.4	2.31	2, 26	0.1	-0.01		0.9	1.30	2, 17	0.3	
** - Somatic z-score**	-0.05	0.7	1.4	2, 26	0.3							

GABA: Gamma-aminobutyric acid, i.u.: institutional units, BP_ND_: non-displaceable binding potential

* Includes treatment allocation (sham or active intermittent theta burst stimulation)

The *vegetative score* did not correlate with GABA levels in the dACC or the mean BP_ND_ in the hypothalamus, either at baseline or in change measurements (Tables 3 and 4).

Neither were any significant results found when accounting for treatment allocation in separate multiple regression analyses of changes in *psychomotor* and *vegetative scores*, with the GABA measures as the dependent variables (Table 4).

The *somatic score* did not correlate with GABA levels in the dACC, either at baseline or in change measurements (Tables 3 and 4). The voxel-wise regression analysis of the baseline *somatic score* and BP_ND_ in the anterior cingulate loop−hypothalamus ROI did not identify any clusters where the measures were related. Similarly, changes in the *somatic score* and in BP_ND_ did not yield any significant clusters (data not shown).

Voxel-wise regression analyses of accelerometry and BP_ND_ in the anterior cingulate loop–hypothalamus ROI also did not identify any clusters where the measures were related (data not shown).

MRS quality metrics were found to be satisfactory according to consensus criteria [45]: the SNR was 214 (SD: 36), and the FWHM was 6.5 Hz (SD: 1.0). The average relative residuals were 19.5 (SD: 7.7) for the OFF spectra and 7.9 (SD: 3.1) for the DIFF spectra.

## Discussion

This is the first study to examine whether psychomotor retardation and vegetative dysfunction during depressive episodes are associated with GABA in the anterior cingulate loop and hypothalamus. However, the study was unable to establish such a relationship.

Although findings have been inconsistent, a growing body of evidence suggests GABA dysfunction during depressive episodes, potentially with greater relevance in melancholic symptom profiles [2, 46]. Melancholic depression, as assessed through psychomotor retardation, seems to predict antidepressive effectiveness of ECT [47, 48]. Given the long-standing hypothesis that GABA plays a role in the mechanism of ECT, this may indicate a link between melancholic features and GABA [49]. The phenotypically related disorder of catatonia is also suggested to involve GABA dysfunction in the basal ganglia, as evidenced by the rapid effect of benzodiazepine treatment [50]. A case series study has described a fast and complete response to lorazepam for depressive symptoms in melancholic patients, suggesting a closer relationship between depression, catatonia and GABA than the current understanding [51].

However, very few studies have specifically investigated GABA in relation to melancholic features. An early study found that unipolar melancholic patients had lower GABA levels in cerebrospinal fluid compared to other depressed patients [12]. This finding was replicated in a study examining plasma GABA, which found reduced levels in melancholic patients compared to healthy controls [11]. A study using transcranial magnetic stimulation to assess motor cortex excitability found no significant change in an index of GABA_A_-receptor transmission in melancholic depression, but reported a higher resting motor threshold in melancholic than in undifferentiated depression [52]. The motor threshold is regarded as a measure of net cortical excitability; while mainly influenced by excitatory mechanisms, it is also affected by tonic GABA-mediated inhibition [53].

Using MRS to assess brain GABA levels and [^11^C]flumazenil PET to measure GABA_A_-receptor availability, our study could not corroborate these earlier findings. Although both psychomotor retardation and vegetative dysfunction are considered typical melancholic features [9, 10], there have been difficulties in empirically establishing which symptoms differentiate melancholia from other depressive episodes. In an extensive review, psychomotor retardation and mood non-reactivity were the only clinical variables that consistently differentiated melancholia from non-melancholic depression across studies [54]. The broader and less specific nature of vegetative symptoms may therefore have contributed to the absence of significant associations in the present study.

Further, the patients in this study were not diagnosed with melancholic depression, although they experienced a significant burden of depressive symptoms. The relatively low baseline scores for psychomotor and vegetative symptoms restricted variability. Consequently, the delta values of symptom scores between time points showed a similar restriction of range, which reduced the strength of the correlations that could be observed.

The difficulties in defining melancholia are closely linked to the ongoing debate over whether melancholia represents a distinct clinical entity or merely is a marker of depression severity [55, 56]. The binary model emphasises the preferential response of melancholia to physical therapies, suggesting a separate neurobiological underpinning [57]. In contrast, current diagnostic systems follow the unitary model, where melancholia is viewed as a specifier for a depressive episode diagnosis [9, 10]. According to the unitary view, the intensity of the melancholic symptoms forms the basis for this specification. This implies that the symptoms may emanate from the same biological mechanism, regardless of their severity, even in patients who do not fulfil the criteria for the melancholic specifier. If the present study had identified associations between GABA and psychomotor or vegetative symptoms across the study sample, this would have supported the unitarian hypothesis of melancholia. Conversely, if melancholia instead represents a categorically distinct condition, as proposed by the binary view, assessing milder forms of psychomotor or vegetative symptoms dimensionally may not adequately capture the relevant biological phenotype.

A further consideration concerns the selection of brain region for MRS. While the dACC was a theoretically relevant region suitable for single-voxel MRS, other regions, including the dorsolateral prefrontal cortex and the premotor and supplementary motor areas, may also contribute to psychomotor symptoms [17, 23]. Accordingly, the absence of an association between melancholic features and dACC GABA does not preclude alterations in GABA levels elsewhere within a broader neural circuitry underlying psychomotor disturbances.

There are also technical constraints when investigating in vivo GABA transmission in the brain. Even if there is a dysfunction in GABA neurotransmission, it is not certain that this will result in a measurable change in GABA levels or GABA_A_-receptor binding potential. MRS provides a measurement of inhibitory tone but cannot detect changes in phasic inhibitory transmission, which occurs at micromolar levels [58]. Flumazenil, used as a proxy for GABA_A_-receptor availability in PET, has an affinity for all α-subunits of the GABA_A_-receptor [59], meaning that changes in the distribution of subunits cannot be captured. Post-mortem findings indicate an altered expression of GABA_A_-receptor subunits in the frontal cortex tissue of individuals with depression [60, 61]. If depressive episodes or depressive subtypes are indeed associated with such changes, [^11^C]flumazenil PET will not be able to identify them.

### Limitations

In this study, psychomotor retardation was assessed using the CAINS expression subscale. Originally developed for schizophrenia spectrum disorders, the CAINS has also been validated for use in depressive episodes [27, 28]. Importantly, we specifically used the expression subscale, which captures observable expressive and motor-related features such as facial expression, vocal prosody, gestures, and speech, all of which overlap with classical manifestations of psychomotor retardation in depression. However, the instrument was not specifically developed or validated for melancholic depression, and may not fully capture the broader phenomenology of psychomotor retardation in depressive states. Recent network analysis work has further highlighted substantial overlap between psychomotor slowing and negative symptoms, suggesting that these domains may share partially overlapping behavioural and neurobiological mechanisms [62]. Consequently, it remains possible that the construct assessed in the present study partly reflected negative symptom dimensions with neurobiological mechanisms distinct from GABA dysfunction. This may have reduced construct specificity and contributed to the null findings.

CORE, a psychomotor-based measure of melancholia [63], has been shown to correlate with accelerometry in elderly depressed inpatients [64]. While this suggests accelerometry measurements reflects melancholic psychomotor retardation, it remains uncertain whether these findings can be generalised to the participants in this study, who were both younger and outpatients.

Sleep disturbances were assessed using items derived from the PSQI [29], focusing on terminal insomnia. However, these items may not accurately reflect this sleep pattern, as initial and middle insomnia could also influence the overall score.

Appetite loss was assessed using a single MADRS-S item rather than a dedicated multi-item measure of appetite disturbance [30], which may have limited the precision and sensitivity of the assessment. However, the item was incorporated only as one component of a broader composite measure of vegetative symptoms. In addition, the measure did not capture weight loss, a clinically important vegetative feature often associated with more severe melancholic depression.

An additional limitation is the potential confounding influence of concomitant pharmacotherapy on GABA measures. Although medication remained stable throughout the study, antidepressants, mood stabilisers, and antipsychotics may modulate the GABA system to varying degrees [65, 66]. Such medication-related effects may have increased inter-individual variability in the MRS and PET measures, thereby reducing the sensitivity to detect associations between melancholic features and GABA levels or GABA_A_-receptor availability.

Finally, as the study was conducted within the context of an iTBS intervention trial, treatment-related effects on both symptoms and GABA measures cannot be excluded. Although additional analyses adjusted for treatment allocation, the potential influence of treatment effects should be considered when interpreting the longitudinal findings.

## Conclusion

In this MRS and PET study, no associations were found between psychomotor and vegetative symptoms during depressive episodes and GABA levels in the dACC, or GABA_A_-receptor availability in the anterior cingulate loop and hypothalamus. The lack of associations may be attributed to several factors, other than an actual absence of a relationship between melancholic features and GABA. Accordingly, future research on the role of GABA dysfunction in the pathophysiology of depression should ideally focus on patients exhibiting more pronounced psychomotor and vegetative symptoms and extend investigations to additional cortical regions implicated in these processes.

## Data Availability

Data cannot be provided due to ethical and legal limitations. For additional inquiries, please contact the corresponding author.

## Abbreviations

ACC: Anterior cingulate cortex
BP_ND_: Non-displaceable binding potential
CAINS: Clinical Assessment Interview for Negative Symptoms
dACC: Dorsal anterior cingulate cortex
ECT: Electroconvulsive therapy
FWHM: Full width at half maximum
GABA: Gamma-aminobutyric acid
MADRS-S: Montgomery Åsberg Depression Rating Scale – self-rated
MEGA-PRESS: Mescher-Garwood spectral editing technique within a point-resolved spectroscopy sequence
MINI: Mini International Neuropsychiatric Interview
MNI: Montreal Neurological Institute
MRI: Magnetic resonance imaging
MRS: Magnetic resonance spectroscopy
PET: Positron emission tomography
pFWE: Family-Wise Error corrected p-value
PSQI: Pittsburgh Sleep Quality Index
SNR: Signal-to-noise ratio
SPM: Statistical Parametric Mapping
